# Dietary Intake and Symptom Severity in Women with Fecal Incontinence

**DOI:** 10.1007/s00192-024-05776-6

**Published:** 2024-04-24

**Authors:** Uduak U. Andy, Jeniffer Iriondo-Perez, Benjamin Carper, Holly E. Richter, Keisha Y. Dyer, Maria Florian-Rodriguez, G. Sarah Napoe, Deborah Myers, Michele O’Shea, Donna Mazloomdoost, Marie G. Gantz, the Pelvic Floor Disorders Network

**Affiliations:** 1 Department of Obstetrics and Gynecology, University of Pennsylvania, 3737 Market Street, 12Th Floor, Philadelphia, PA 19104 USA; 2 Social, Statistical & Environmental Sciences, RTI International, Research Triangle Park, Durham, NC USA; 3 Department of Obstetrics and Gynecology, University of Alabama at Birmingham, Birmingham, AL USA; 4 Department of Obstetrics and Gynecology Kaiser Permanente, San Diego, CA USA; 5 Department of Obstetrics and Gynecology, University of Texas Southwestern Medical Center, Dallas, TX USA; 6 Department of Obstetrics, Gynecology and Reproductive Sciences, University of Pittsburgh School of Medicine, Pittsburgh, PA USA; 7 Department of Obstetrics and Gynecology, Brown University, Providence, RI USA; 8 Department of Obstetrics and Gynecology, Duke University Medical Center, Durham, NC USA; 9 The Eunice Kennedy Shriver National Institute of Child Health and Human Development, National Institutes of Health, Bethesda, MD USA

**Keywords:** Fecal incontinence, Diet, Fat, Fiber, Dietary assessment

## Abstract

**Introduction and Hypothesis:**

The goal of this study was to determine whether dietary fat/fiber intake was associated with fecal incontinence (FI) severity.

**Methods:**

Planned supplemental analysis of a randomized clinical trial evaluating the impact of 12-week treatment with percutaneous tibial nerve stimulation versus sham in reducing FI severity in women. All subjects completed a food screener questionnaire at baseline. FI severity was measured using the seven-item validated St. Mark’s (Vaizey) FI severity scale. Participants also completed a 7-day bowel diary capturing the number of FI-free days, FI events, and bowel movements per week. Spearman’s correlations were calculated between dietary, St. Mark’s score, and bowel diary measures.

**Results:**

One hundred and eighty-six women were included in this analysis. Mean calories from fats were 32% (interquartile range [IQR] 30–35%). Mean dietary fiber intake was 13.9 ± 4.3 g. The percentage of calories from fats was at the higher end of recommended values, whereas fiber intake was lower than recommended for adult women (recommended values: calories from fat 20–35% and 22–28 g of fiber/day). There was no correlation between St. Mark’s score and fat intake (r = 0.11, p = 0.14) or dietary fiber intake (r = −0.01, *p* = 0.90). There was a weak negative correlation between the number of FI-free days and total fat intake (r = −0.20, *p* = 0.008). Other correlations between dietary fat/fiber intake and bowel diary measures were negligible or nonsignificant.

**Conclusion:**

Overall, in women with moderate to severe FI, there was no association between FI severity and dietary fat/fiber intake. Weak associations between FI frequency and fat intake may suggest a role for dietary assessment in the evaluation of women with FI.

## Introduction

Fecal incontinence (FI), also known as accidental bowel leakage (ABL), is a debilitating condition that affects 9–19% of community dwelling women [1]. First-line management for FI includes conservative management techniques such as dietary modifications. Existing studies show that women with ABL are very interested in dietary modifications [2–4] and seek out advice on how to manage their diet to prevent FI. Unfortunately, the diet recommendations currently available for women are empirical, with limited supporting evidence [5].

Relatively few studies have investigated the role of diet in women with ABL. The few studies that exist suggest that compared with healthy controls, low dietary fiber intake may be associated with developing FI [6–8]; however, there are no data assessing the association of FI severity with fiber intake in women with FI. Similarly, there are scant data examining the role of dietary fat in FI severity despite the results of qualitative studies that reveal that women with FI commonly modify their fat intake to control their symptoms [2, 3]. This gap in knowledge likely contributes to patients’ reports of receiving inadequate therapeutic advice from their physicians regarding dietary changes to improve FI symptoms [4].

The goal of this study was to determine whether specific components of dietary intake are associated with FI severity. We hypothesize that dietary fiber and fat intake might be associated with FI severity.

## Materials and Methods

This was a planned supplemental analysis of the previously published NeurOmodulatTion for Accidental Bowel Leakage (NOTABLe) trial, a randomized clinical trial evaluating the impact of 12-week treatment with percutaneous tibial nerve stimulation (PTNS) versus sham in reducing FI severity in women [9]. The NOTABLe trial population consisted of women aged ≥ 18 with moderate to severe ABL for ≥ 3 months and inadequate symptom control from supervised pelvic muscle training and constipating medications. Women with extremes of stool type (uncontrolled diarrhea or severe constipation), those with anatomical compromise to the anus or rectum, and those with known contraindications for PTNS were excluded [9].

The primary study was conducted at 9 US clinical sites of the National Institutes of Health Pelvic Floor Disorders Network with the approval of a Data and Safety Monitoring Board and the University of Pittsburgh institutional review board (NCT 03278613). All clinical trial participants provided written informed consent. The trial included a 4-week run-in phase prior to randomization to 12 weekly sessions of PTNS versus sham stimulation. Dietary intake data and baseline FI symptom severity were collected prior to the run-in phase. Participants also completed a 7-day bowel diary during week 1 of the run-in phase.

Dietary data were collected using a food screener questionnaire. The Food Screener is a validated one-page self-administered tool with two sections: a 17-item Meat/Snacks section designed to capture dietary fats and a ten-item Fruit/Vegetable section to capture fruit, vegetable, and fiber intake. The screener also captures liquid intake [10]. The instrument generates point estimates of total and saturated fat (g), percentage of calories from fat, daily fruit and vegetable servings, and fiber (g). The Food Screener has been validated against the 1995 Block 100-item Food Frequency Questionnaire and has been used in clinical and research settings to determine usual dietary intake [10]. A single question (dichotomous, yes/no) was added to the Food Screener to assess fiber supplement use. FI severity was measured using the seven-item validated St. Mark’s (Vaizey) FI severity scale. The scale assesses the type and frequency of anal incontinence, alteration in lifestyle from incontinence including the use of pads or plugs, the need to change underwear, the use of antidiarrheal medication, and the ability to defer defecation for 15 min. Total scores range from 0 to 24 with higher scores indicating greater symptom severity [11]. The 7-day bowel diary completed during week 1 of the run-in phase was reviewed to capture pertinent measures, including the number of FI-free days, the number of FI episodes, the number of urge-associated FI episodes, and the number of bowel movements per week.

Descriptive statistics were presented as proportions for categorical variables or as means (standard deviations) or medians (interquartile ranges) for continuous variables, as appropriate.

Spearman’s correlations were calculated between dietary intake with St. Mark’s score and with bowel diary measures. Given that the aim of the study was to assess the association between dietary intake and FI severity, the entire group was analyzed as a single cohort and the analyses were not stratified by treatment group (PTNS versus sham). Observed significant correlations were interpreted as follows: < 0.3 was considered low, between 0.3 and 0.5 was considered moderate, and ≥ 0.5 was considered high [12]. Statistical analyses were conducted using SAS version 9.4 (SAS Institute Inc., Cary, NC, USA).

## Results

Enrollment for the NOTABLe trial occurred between 9 February 2018 and 24 September 2019. One hundred and ninety-nine women entered the run-in. For this analysis, the 186 women who completed the run-in phase and had complete diary data (defined as a minimum of 3 consecutive days during week 1 of the run-in phase) as well as dietary data and FI severity data available were included. Baseline demographic and clinical characteristics of the women included in this analysis are presented in Table 1. Mean age and body mass index (BMI) were 63.8 ± 11.5 years and 29.4 ± 6.6 kg/m^2^ respectively. The mean baseline St. Mark’s score was 17.8 ± 2.6 and mean baseline FI episodes per week was 7.9 ± 7.8 indicating moderate to severe FI. Fiber supplementation was reported by 75 out of 180 (41.7%) of participants.

**Table 1:** Baseline demographics and clinical characteristics

Characteristic	Category	Total (*N* = 186)
Age (years): mean (SD) [minimum, maximum]		63.8 (11.5) [29.0, 90.0]
Race, *p*/*N* (%)	American Indian or Alaska Native	2/186 (1.1)
	Asian	2/186 (1.1)
	Black or African American	23/186 (12.4)
	Native Hawaiian or Other Pacific Islander	2/186 (1.1)
	White	150/186 (80.6)
	Unknown/not reported	7/186 (3.8)
Ethnicity, *n*/*N* (%)	Hispanic/Latina	18/186 (9.7)
	Not Hispanic/Latina	166/186 (89.2)
	Unknown/not reported	2/186 (1.1)
Primary language, *n*/*N* (%)	English	179/186 (96.2)
	Spanish	7/186 (3.8)
Education, *n*/*n* (%)	Some college or greater	131/185 (70.8)
	No college education	54/185 (29.2)
Insurance status, *n*/*N* (%)	Private/HMO	68/186 (36.6)
	Medicare/Medicaid	49/186 (26.3)
	Both private and Medicare/Medicaid	48/186 (25.8)
	Other/none	21/186 (11.3)
Body mass index (kg/m^2^): mean (SD) [minimum, maximum]		29.4 (6.6) [18.0, 55.0]
Body mass index, *n*/*N* (%)	< 25 kg/m^2^	46/184 (25.0)
	25—29.9 kg/m^2^	58/184 (31.5)
	≥ 30 kg/m^2^	80/184 (43.5)
Presence of anal sphincter squeeze, *n*/*N* (%)		165/184 (89.7)
Vaginal deliveries, median [Q1, Q3]		2.0 [1.0, 3.0]
Cesarean deliveries, median [Q1, Q3]		0.0 [0.0, 0.0]
Menopausal status, *n*/*N* (%)	Pre-menopausal	17/186 (9.1)
	Post-menopausal	158/186 (84.9)
	Not sure	11/186 (5.9)
Currently using estrogen, *n*/*N* (%)		52/186 (28.0)
Current smoker, *n*/*N* (%)		15/186 (8.1)
Previous ABL surgery, *n*/*N* (%)		10/186 (5.4)
Previous anal/rectal surgery, *n*/*N* (%)		34/186 (18.3)
Previous UI surgery, *n*/*N* (%)		44/186 (23.7)
Previous POP surgery, *n*/*N* (%)		45/186 (24.2)
Hysterectomy, *n*/*N* (%)		84/186 (45.2)
Taking fiber supplements, *n*/*N* (%)		75/180 (41.7)
Bristol Stool Type, *n*/*N* (%)	Type 2—Sausage-shaped but lumpy	19/186 (10.2)
	Type 3—Like a sausage but with cracks on its surface	24/186 (12.9)
	Type 4—Like a sausage or snake, smooth and soft	47/186 (25.3)
	Type 5—Soft blobs with clear-cut edges	40/186 (21.5)
	Type 6—Fluffy pieces with ragged edges, a mushy stool	56/186 (30.1)
Pain/discomfort in abdomen in last 3 months, *n*/*N* (%)	Less than once per week	115/186 (61.8)
	At least once per week	71/186 (38.2)
Pain/discomfort 6 months or longer, *n*/*N* (%)		91/186 (48.9)
Diagnosed with IBS, *n*/*N* (%)		39/186 (21.0)
Frequency of loose/mushy/watery stools in last 3 months, *n*/*N* (%)	Never or rare	65/186 (34.9)
	Sometimes	39/186 (21.0)
	Often/most of the time/always	82/186 (44.1)
St. Mark’s score (start of run-in), mean (SD) [minimum, maximum]		17.8 (2.6) [12.0, 24.0]
Bowel movements per week (start of run-in), mean (SD) [minimum, maximum], no		12.8 (8.1) [0.0, 45.5]
Bowel movements with urgency per week (start of run-in), mean (SD) [minimum, maximum], no		6.2 (5.9) [0.0, 35.0]
Accident-free days per week (start of run-in), mean (SD) [minimum, maximum], no		3.1 (2.1) [0.0, 7.0]
Leaks per week (start of run-in), mean (SD) [minimum, maximum], no		7.9 (7.8) [0.0, 52.0]
Leaks with urgency per week (start of run-in), mean (SD) [minimum, maximum], no		3.4 (4.4) [0.0, 35.0]

*ABL* accidental bowel leakage, *UI* urinary incontinence, *POP* pelvic organ prolapse, *IBS* irritable bowel syndrome

Dietary intake data are listed in Table 2. Mean total and saturated fat intake was 86.8 ± 20.7 g and 21.6 ± 7.6 g respectively and the median percentage of calorie consumption from fats was 32% [IQR 30–35%]. Mean fruit and vegetable servings were 3.3 ± 1.6 and mean fiber intake was 13.9 ± 4.3 g. Dietary intake reported by women with FI was higher in percentage of calories from fat and lower in fiber than recommended daily intakes (recommended values: calories from fat 20–35%; five fruit and vegetable servings/day; and 22–28 g of fiber/day).

**Table 2:** Baseline dietary characteristics

Outcomes	Overall
Meat/Snack score, *n*	174
Mean (SD) [minimum, maximum]	17.9 (8.6) [0.0, 52.0]
Median (p25, p75)	17.0 (13.0, 22.0)
Total fat (g), *n*	174
Mean (SD) [minimum, maximum]	86.8 (20.7) [43.9, 168.7]
Median (p25, p75)	84.7 (75.1, 96.7)
Saturated fat (g), *n*	174
Mean (SD) [minimum, maximum]	21.6 (7.6) [5.9, 51.7]
Median (p25, p75)	20.9 (17.3, 25.3)
Percent fat, *n*	174
Mean (SD) [minimum, maximum]	32.8 (5.2) [22.1, 53.3]
Median (p25, p75)	32.3 (29.9, 35.3)
Dietary cholesterol (g), *n*	174
Mean (SD) [minimum, maximum]	211.9 (71.2) [65.4, 471.0]
Median (p25, p75)	205.8 (166.8, 244.8)
Fruit/Vegetable/Bean screener score, *n*	175
Mean (SD) [minimum, maximum]	14.1 (5.9) [1.0, 36.0]
Median (p25, p75)	14.0 (10.0, 17.0)
Fruit/Vegetable screener score, *n*	176
Mean (SD) [minimum, maximum]	11.0 (4.4) [0.0, 26.0]
Median (p25, p75)	11.0 (8.0, 14.0)
Fruit/Vegetable servings, *n*	176
Mean (SD) [minimum, maximum]	3.3 (1.6) [0.8, 8.8]
Median (p25, p75)	3.3 (2.2, 4.4)
Vitamin C (mg), *n*	175
Mean (SD) [minimum, maximum]	94.2 (37.9) [6.7, 234.1]
Median (p25, p75)	90.7 (67.0, 118.6)
Magnesium (mg), *n*	175
Mean (SD) [minimum, maximum]	234.5 (66.6) [79.1, 471.5]
Median (p25, p75)	227.9 (189.1, 279.7)
Dietary fiber (g), *n*	175
Mean (SD) [minimum, maximum]	13.9 (4.3) [4.1, 30.0]
Median (p25, p75)	13.8 (10.8, 16.0)
Potassium (mg), *n*	175
Mean (SD) [minimum, maximum]	2,325.4 (657.6) [793.0, 4,700.6]
Median (p25, p75)	2,257.8 (1,881.4, 2,772.2)

There were no significant correlations between St. Mark’s score and fat intake (r = 0.11, *p* = 0.14), fruit/vegetable servings (r = 0.06, *p* = 0.45), or dietary fiber intake (r = −0.01, *p* = 0.90; Table 3). There was a weak negative correlation between the number of FI-free days and total fat intake (r = −0.20, *p* = 0.008) and fruit/vegetable servings (−0.21, *p* = 0.006; Table 4). Other correlations between dietary intake and bowel diary measures were negligible or nonsignificant (data not shown).

**Table 3:** Correlation between dietary characteristics and St. Mark’s score

Label	*n*	Spearman value	*p* value
Meat/Snack score	174	0.1112	0.1442
Total fat (g)	174	0.1112	0.1442
Saturated fat (g)	174	0.1112	0.1442
Percentage fat	174	0.1112	0.1442
Dietary cholesterol (g)	174	0.0950	0.2125
Fruit/Vegetable/Bean screener score	175	−0.0099	0.8970
Fruit/Vegetable screener score	176	0.0575	0.4488
Fruit/vegetable servings	176	0.0575	0.4488
Vitamin C (mg)	175	−0.0051	0.9466
Magnesium (mg)	175	−0.0051	0.9462
Dietary fiber (g)	175	−0.0099	0.8970
Potassium (mg)	175	−0.0075	0.9216

The Meat/Snack score and the fat and cholesterol point estimates are derived from the 17-item Meat/Snack section of the Food Screener. The Fruit/Vegetable/Bean screener score and the fruit, vegetable, fiber and vitamin estimates are derived from the ten-item Fruit/Vegetable section of the Food Screener

**Table 4:** Correlation of dietary characteristics and mean number of fecal incontinence-free days per week

Label	*n*	Spearman value	*p* value
Meat/Snack score	172	−0.202	0.0079
Total fat (g)	172	−0.202	0.0079
Saturated fat (g)	172	−0.202	0.0079
Percent fat	172	−0.202	0.0079
Dietary cholesterol (g)	172	−0.195	0.0105
Fruit/Vegetable/Bean screener score	173	−0.135	0.0772
Fruit/Vegetable screener score	174	−0.210	0.0055
Fruit/vegetable servings	174	−0.210	0.0055
Vitamin C (mg)	173	−0.127	0.0970
Magnesium (mg)	173	−0.108	0.1573
Dietary fiber (g)	173	−0.135	0.0772
Potassium (mg)	173	−0.115	0.1313

The Meat/Snack score and the fat and cholesterol point estimates are derived from the 17-item Meat/Snack section of the Food Screener. The Fruit/Vegetable/Bean screener score and the fruit, vegetable, fiber, and vitamin estimates are derived from the ten-item Fruit/Vegetable section of the Food Screener

## Discussion

Dietary advice is typically part of the first line of treatment for women with FI; however, data on the contribution of diet intake and modifications on FI symptoms are lacking. In this ancillary analysis of a large multicenter randomized trial, we sought to assess the relationship between dietary intake and bowel symptoms in women with FI. Although an association between FI severity and dietary fat/fiber intake was not found, weak associations were noted between greater FI frequency and increasing fat intake. The findings of this study add to the limited data on dietary intake in women with FI and may suggest a role for dietary assessment in the evaluation of women with FI.

The associations found between bowel symptoms and fat intake were weak and of unclear clinical significance; however, they are not surprising. In qualitative studies that examine self-care practices of women with FI, women report that they commonly modify their fat intake to control FI symptoms [3]. Mechanistically, a high fat diet could cause FI by causing stools to be too hard or too liquid, both of which are independent risk factors for FI in large epidemiological studies [13–16]. Fat intake has also been shown to decrease gastrointestinal motility and negatively affect the function of the neurons in the enteric nervous system [17, 18]. Although these studies suggest that dietary fat might be able to alter stool consistency and/or gastrointestinal motility and potentially contribute to FI, existing studies investigating the diet of women with FI are limited. Even though the current findings add to the available data on the relationship between dietary fat and FI, additional studies are needed to examine this relationship more robustly.

There was no association between dietary fiber intake and FI severity within our cohort. Prior studies have suggested that there might be an inverse relationship between dietary fiber intake and development of FI [6, 8]; however, others have not reported an association between intake of food with varying amounts of fiber and FI severity. It is possible that given that the dietary fiber intake amongst our cohort was very low with a narrow distribution, there was a limited ability to detect a correlation. Our cohort, though, is similar to other reported cohorts of women with FI with lower-than-average fiber consumption. Prior studies reporting on diet intake of women with FI found a mean daily dietary fiber intake of 13–15 g, which is similar to our cohort [19, 20]. Thus, prospective intervention studies that assess the impact of varying intakes in dietary fiber are likely needed to fully assess the relationship between dietary fiber and FI severity.

Documentation of usual dietary intake may be helpful in the workup and management of women with FI, especially if noted to be outside the recommended ranges for proportion of fat, fat type, and fiber intake. Additionally, assessing dietary intake enables the identification of potential triggers and provides diet modification opportunities that may improve symptoms. Currently, dietary advice provided for women with FI is usually empirical, i.e., increase dietary fiber and decrease fat. As Bliss et al. noted, even in the absence of change in overall nutrition profile, managing FI may require increased or decreased consumption of specific individual foods [19]. Assessing current usual diet intake may provide an opportunity to personalize the dietary advice provided and improve the current conservative management strategies for treating FI. In this study, a simple food questionnaire was used to ascertain usual dietary intake. Other methods such as food diaries and 24-h dietary recalls have also been employed to measure dietary intake, although they require more effort [21].

Strengths of this study include use of a large, well-characterized, multicenter cohort of women with moderate to severe FI from a randomized controlled trial. A validated food screener and validated FI severity questionnaires that have been utilized in both clinical and research settings to document diet intake and bowel symptoms were used. There are also several limitations. The screener allows point estimates of food categories such as meat/snacks and fruits/vegetables, but only assesses a limited profile of dietary intake and does not provide granular details about individual foods that would allow an assessment of potential triggers. Although St. Mark’s scale has been extensively validated and is widely accepted as a measure of FI severity, there are limitations in that it may not adequately reflect the severity of FI in some patients, such as those with passive FI, and may not capture the total patient perspective on their symptoms [22]. Women completed the food screener during the run-in phase of the randomized controlled trial, which may affect their report of dietary intake. We only included a single nonspecific question about the use of fiber supplementation and thus we are unable to comment on the varied types of fiber supplements and the different effects they may have on bowel function. We did not collect data on history of cholecystectomy or colon surgery, which can impact FI symptoms and dietary absorption. Finally, there are limitations inherent to the primary study, including that this was a cohort of women seeking care for FI at urogynecology clinics and thus these findings may not be generalizable to a different population, and comparison with a control group without FI is beyond the scope of this paper [9].

In conclusion, this study adds to the current literature on dietary intake in women with FI. A weak and unclear clinically significant association was noted between FI and fat intake that may suggest a role for dietary assessment in the evaluation and management of women with FI.

## Data Availability

The data for the trial is publicly available through the NICHD Data and Specimen Hub.
